# Differences in the Portrayal of Female and Male Scientists in College Chemistry Textbooks

**DOI:** 10.1021/acs.jchemed.4c01365

**Published:** 2025-04-09

**Authors:** Peyton T. Fair, Melanie R. Nilsson

**Affiliations:** † Department of Biology, McDaniel College, Westminster, Maryland 21157, United States; § Department of Chemistry, McDaniel College, Westminster, Maryland 21157, United States

**Keywords:** Chemistry Textbooks, Recommendation Letters, Teaching Evaluations, Gender Bias, Marie Curie, Linus Pauling

## Abstract

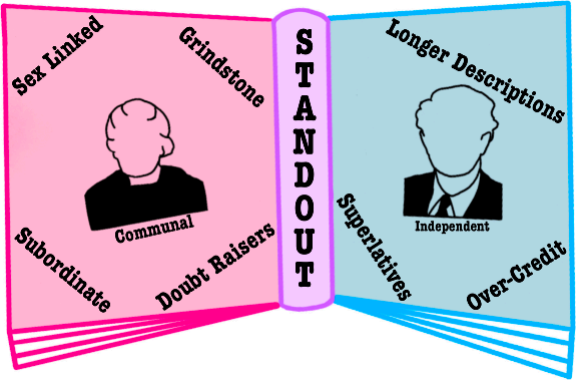

This study examines the portrayal of one female and one male scientist, Marie Curie and Linus Pauling, in 10 US General Chemistry textbooks published between 2016 and 2020. The language in the textbooks was analyzed using methods previously developed for the study of letters of recommendation. Textbook descriptions of Marie Curie are shorter and contain a higher frequency of sex-linked words, subordinate language, grindstone terms, doubt raisers, and communal attributes. Descriptions of Linus Pauling are longer and characterized by a higher frequency of references that highlight his independence. The frequency of standout words (words that indicate exceptional attributes) is the same for Curie and Pauling, but more superlatives and repetition of standout words are used with Linus Pauling. The accuracy of the statements made in the textbooks was also examined, which is a unique avenue of investigation that is often not possible in studies of performance evaluations. The textbook inaccuracies for Linus Pauling consistently give him more credit than he is due, while those for Marie Curie are mixed and often undermine her accomplishments. The language and characterization of Marie Curie and Linus Pauling convey different expectations for female and male scientists, which may impact entry, retention, and promotion in the discipline.

## Introduction

Textbooks reflect what established practitioners in a discipline believe to be important and, therefore, provide insight into expected professional norms. They are also used as tools within the discipline. Teachers use textbooks to plan courses and design in-class activities, while students use textbooks to review concepts and practice skills. Thus, although textbooks are not typically read in their entirety, there are many opportunities for the content of textbooks to define, shape, and reinforce values and expectations within a discipline.^1^

General Chemistry textbooks were previously shown to contain significant differences in the number of male and female scientists represented in the texts.^2^ The purpose of this study is to explore the ways in which male and female scientists are portrayed in the textbooks. The goal is to move beyond “who” is included to understand “how” the individuals in textbooks are depicted.

This study was inspired by prior research in which differences in the portrayal of male and female candidates were observed in letters of recommendation,^3−8^ performance evaluations,^9^ teaching evaluations,^10,11^ and the way guest speakers are introduced.^12^ These differences manifest in two common ways: identical characteristics being viewed differently depending on the assumed gender identity^10,13,14^ and differences in how females and males are characterized.^3,4,6,9,11,15^ In both cases, the differences may perpetuate gender-based stereotypes and limit inclusion and advancement in STEM fields.

The textbook descriptions of two renowned scientists, Marie Curie and Linus Pauling, were chosen for comparison. Textbook language was analyzed using methods previously developed for the study of recommendation letters and performance evaluations. The accuracy of the statements made about the two scientists, particularly those related to the attribution of credit, was also evaluated. The objective was to determine whether textbooks convey different norms or expectations for female versus male scientists.

## Materials and Methods

See the Supporting Information (SI) for additional details.

### Selection of Scientists

Textbooks were selected using a previously described method^2^ and included 10 General Chemistry textbooks (two-semester, majors-level) published between 2016 and 2020.^16−25^ Marie Curie was chosen for this study because she is the most frequently indexed female scientist in these textbooks.^2^ Since textbooks often cite Nobel prizes as a metric of accomplishment, this parameter was used to identify a male scientist of similar status. Marie Curie won two Nobel prizes (chemistry and physics), and Linus Pauling is the only other scientist in these General Chemistry textbooks to also win two Nobel prizes (chemistry and peace).

Marie Curie is one of only 12 female STEM professionals mentioned across the 10 textbooks (and one of only five to appear in more than one textbook),^2^ and therefore, the descriptions of her are likely to significantly shape perceptions about females in the discipline. Since Linus Pauling is one of 348 male STEM professionals in the 10 textbooks,^2^ the portrayal of him may not carry the same weight in defining disciplinary expectations of males. However, Pauling is one of only eight males included in all 10 textbooks,^2^ and this breadth of exposure is likely to elevate the impact of descriptions of him.

### Identification of Passages

The index was used to locate passages pertaining to Marie Curie and Linus Pauling. However, it quickly became apparent that incorrect indexing was common. Therefore, four pages before and four pages after each indexed page were examined to identify references to the scientists. If an unindexed page about the scientist was identified in this manner, then four pages before and after each unindexed page were examined, and the process repeated until no more references were observed within a plus or minus four-page interval.

### Transcripts

Each page with information about Marie Curie and Linus Pauling was used in this study, even if the page was unindexed. Sentences containing their names or respective pronouns (singular or plural) were transcribed into Microsoft Word using a single-spaced Calibri 12-point font. Any figure numbers or equations that were directly part of a sentence that included the name or pronoun of the scientist were included. The transcripts for all 10 textbooks were combined for each scientist to provide a holistic perspective across all texts.

### Length

Length (word and line count) was determined using Microsoft Word file statistics.

### Sex-Linked Terms

Sex-linked terms were defined as all words that imply the sex of the scientist (e.g., woman, father, her, gentleman). The examination of sex-linked terms applies binary definitions of sex and was performed to compare the textbooks with prior studies. However, we recognize that variations in biological sex exist and there are over 50 gender identities.^26,27^ Thus, this method does not purport to explicitly define the biological sex or gender identity of the individuals but rather to reflect the sex or gender identity most likely perceived by the reader.

### Subordinating Language

Subordinating language was operationally defined as the use of language that may downgrade the status of the scientist. Examples include the use of alternate titles (e.g., Ms., Mr.) in lieu of an academic title (e.g., Dr., Professor), references to subordinate positions, or the use of only the first name.

### Agentic and Communal Proxies

Agentic traits relate to a person’s agency, which describes an individual’s ability to generate a desired result (e.g., independence, self-reliance, assertiveness). Communal attributes, in contrast, place emphasis on the care and concern for others (e.g., helpful, caring, sympathetic). Agentic and communal adjectives are uncommon in the transcripts. However, since agency is individualistic and communality relates to other people, we used the number of mentions of the scientist’s name as a proxy for agency and the number of mentions of other people as a communal indicator. An additional measure of communal orientation was provided by counting the number of familial terms in each transcript (e.g., wife, son, family).

### Standout, Grindstone, and Doubt Raiser Language

Descriptions that correspond to the categories of standout, grindstone, and doubt raiser were quantified for each scientist. The standout category includes adjectives, superlatives, or phrases that indicate a degree of exceptionality. Grindstone language includes words or phrases that emphasize an input of time or describe activities that are more associated with effort than with skill or innovation. Doubt raisers are words or phrases defined by Trix and Psenka as “including negative language, along with hedges, potentially negative comments, unexplained comments, faint praise, and irrelevancies”.^3^

These descriptors (standout, grindstone, and doubt raiser) were operationally defined to be mutually exclusive and coded within a single document. Published lists of words and word roots for specific categories were used as a guide^3,4^ but did not limit the search. Two coders analyzed the composite transcripts, and the results were averaged. A measure of agreement was calculated by dividing the number of identically coded items by the average number of items coded. Using this measure, the overall agreement across the three categories (standout, grindstone, and doubt raiser) was 96%.

Some descriptors modified the scientist of interest (Curie or Pauling) as well as another person. These descriptors were multiplied by one-half to account for the “sharing” of the descriptor, and these values were used to calculate frequency. See the SI for raw data.

### Nationality

Nationality may be a doubt raiser if considered irrelevant or if it has a potentially negative impact. However, since it is common for textbooks to highlight the global nature of scientific research, nationality was not counted as a doubt raiser but instead was analyzed separately.

### Accuracy

Because historic records exist for each scientist examined in this study, the textbook information was assessed for accuracy. The transcripts for each scientist were compared to records such as Marie Curie’s autobiographical notes,^28^ materials from the Linus Pauling archives,^29^ Nobel prize speeches by the scientists,^30^ and the original published scientific articles. If needed, multiple sources were used, and in some cases, experts were consulted for assistance in translation or interpretation.

Inaccuracies related to the attribution of credit were further examined to assess the impact on the perceived status of the scientist. Attribution inaccuracies were subcategorized as “over-credit” when the scientist was given more credit than warranted, “over-co-credit” when excess credit was given to both the scientist and one or more additional scientists within the same statement, or “under-credit” in which the scientist was not given adequate acknowledgment for their work.

## Results and Discussion

See the Supporting Information for additional details.

### Length

The total word and line counts across the 10 texts are 30% higher for Linus Pauling (words = 1804, lines = 122) compared to Marie Curie (words = 1399, lines = 94). Due to the differences in length, all measured parameters are expressed in terms of frequency of occurrence. See the SI for raw data.

### Sex-Linked Terms and Subordinating Language

Sex-linked terms are used in reference to Marie Curie more than twice as frequently (3.9% of words) compared to Linus Pauling (1.5%). Subordinating language was never used in reference to Linus Pauling, but five instances were found in the Marie Curie transcripts.

### Agentic and Communal Proxies

Linus Pauling’s name is used more frequently (4.2% of words) in comparison to Marie Curie (3.2%). Other people are co-mentioned almost five times more frequently with Marie Curie (2.8% of words) compared to Linus Pauling (0.6%). Familial relationships are 14 times more frequent in the Marie Curie transcripts (1.4% of words) in comparison to Linus Pauling (0.1%). Thus, textbook portrayals of Linus Pauling imply greater agency, while descriptions of Marie Curie reflect more communal attributes and familial terms.

The limited number of people co-mentioned with Linus Pauling prompts the question of the extent to which others were involved in the work for which he is credited. A literature search using SciFinder reveals that Linus Pauling had over 150 different coauthors, including his son, Peter Pauling, with whom he coauthored three papers and a textbook.^31^ Linus Pauling’s wife, Ava Helen, was also a major contributor and, even by Linus Pauling’s own accounts, should have been a corecipient of the Nobel peace prize.^32^ Thus, there were many collaborators and a significant involvement of family members in Linus Pauling’s work, but these individuals are rarely mentioned in the textbooks.

### Standout Descriptors

Standout descriptors occur with the same frequency in the Marie Curie and Linus Pauling transcripts (every 4.4 lines of text). Marie Curie’s standout descriptors primarily relate to Nobel prizes (66%) or the naming of the curie and curium (14%). However, her accomplishments are frequently attenuated. For example, sparse descriptions of the scientific breakthroughs that led to Marie Curie’s Nobel prizes are reminiscent of what Trix and Psenka call “letters of minimal assurance”.^3^ In these instances, “Brevity and generality are clearly seen as reflecting negatively on the applicant” and “leave the reader paying more attention to what is not said as opposed to what is written”.^8^

Linus Pauling’s standout descriptors frequently mention Nobel prizes (38%), but a variety of other forms of praise are included such as “most influential chemist of the twentieth century”,^18^ “genius”,^21^ and “seminal”.^22^ In addition, many modifiers in the Linus Pauling transcripts amplify the impact by using superlatives and a repetition of standout words within the phrases (e.g., “most famous”).^20^

### Grindstone Language

Marie Curie is described with grindstone language three times more frequently (every 4.5 lines of text) in comparison to Linus Pauling (every 15.3 lines). Marie Curie grindstone terms often include derivatives of the words “isolate”, “separate”, or “extract” (56%) and the word “work” (29%). Repetition of grindstone words within a single phrase was observed in the Curie transcripts (e.g., “painstakingly separated”)^22^ but were only counted as one instance of grindstone language. The most common grindstone term used in reference to Linus Pauling was “work” (71%). There was no repetition of grindstone language within a single phrase for Pauling, but many instances (47%) were attenuated by standout adjectives (e.g., “work was a great triumph”).^18^

Trix and Psenka state that the higher percentage of grindstone adjectives for females compared to males amplifies the stereotype that “associates effort with women, and ability with men in professional arenas”.^3^ Brainard notes this stereotype is particularly prevalent in science, and journalists tend to “treat every female scientist they profile as an archetype of perseverance”.^33^

### Doubt Raisers

Doubt raisers occur four times more frequently in the transcripts of Marie Curie (every 4.9 lines of text) in comparison to Linus Pauling (every 18.8 lines). In addition, every textbook that mentions Marie Curie contains at least one doubt raiser, while only 40% of texts include a doubt raiser about Linus Pauling.

Doubt raisers in the Marie Curie transcripts are varied, and examples include (doubt raisers highlighted in italics) the following: “working in an *old shed*”,^22^ “*an older* radioactivity unit is the curie”,^21^ and “she herself *died from aplastic anemia—a disease caused by the inability of bone marrow to produce red blood cells*”.^21^ The majority (62%) of doubt raisers in the Linus Pauling transcripts are caveats to Pauling’s original electronegativity scale (e.g., “Other electronegativity scales have been developed *with slightly different values from those proposed by Pauling*”).^21^

Doubt raisers tend to stand out to the reader and can override other information presented. For example, Madera et al. showed “that the inclusion of even a single doubt raiser—particularly negativity or hedging—was enough to lead to statistically lower evaluations of the applicant”.^5^ Furthermore, the use of “multiple doubt raisers can build upon each other”.^3^ Therefore, the prevalence of doubt raisers pertaining to Marie Curie is notable.

### Nationality

Linus Pauling and Marie Curie are of different nationalities; therefore, both the depiction of nationality and frequency of mention were determined. Linus Pauling is described as American (*n* = 6) or born in Oregon (*n* = 1), while Marie Curie is described primarily as Polish (*n* = 11) and occasionally French (*n* = 2). Marie Curie’s nationality (0.9% of words) is mentioned more than twice as frequently as that of Linus Pauling (0.4%).

The fewer mentions of Linus Pauling’s nationality may indicate the authors assume American is the “norm” or believe Pauling’s nationality is well-known. The more frequent mentions of Marie Curie’s nationality may be to emphasize the international nature of science or because the authors consider her nationality more integral to her life story.

The difference in nationality could, however, impact a reader’s perception of the two scientists. Given the existence of xenophobia in the US,^34^ some readers may perceive Marie Curie’s nationality as a doubt raiser. The difference in nationality of Marie Curie and Linus Pauling may therefore complicate the interpretation of differences in their portrayal, since they differ in both perceived gender and nationality. Further study is needed to understand any potential confounding impact of the nationality.

### Comparison to Letters of Recommendation

The language used in textbook descriptions of Marie Curie and Linus Pauling is very similar to the trends observed in recommendation letters and performance evaluations written for women versus men (see the SI). The ubiquity of gender-specific differences in language among teaching evaluations (written primarily by students) and letters of recommendation and performance evaluations (written primarily by faculty) suggests a common thread. The similarities may reflect dominant cultural norms, or they may be interconnected. For example, the language used to describe male and female scientists in textbooks may shape the language used by students and faculty when writing evaluations. However, future work is needed to determine if any causal or interdependent relationships exist between textbooks and evaluations.

### Accuracy

Every textbook contains mistakes or misrepresentations within the descriptions of Marie Curie and Linus Pauling. Inaccuracies related to the attribution of credit are likely to impact the reader’s perception of the scientist, and these were frequently observed in the Pauling (every 4.9 lines) and Curie (every 3.8 lines) transcripts.

Linus Pauling is over-credited (*n* = 24) and over-co-credited (*n* = 1) but never under-credited. Over-crediting is observed, for example, when textbooks state that Pauling “proposed” the concept of electronegativity and/or devised the “first” electronegativity scale^17,21,22^ (a trio of articles by Jensen highlight electronegativity scales that predate Pauling by over 100 years).^35−37^ Pauling is similarly over-credited for contributions to resonance theory, valence bond theory, protein structure, and sickle-cell disease. For example, one text states that he “proposed the concept of resonance”,^22^ but Pauling himself indicates that Kekulé, Thiele, Arndt, Heisenberg, and others proposed the concept.^38^

Marie Curie is over-credited (*n* = 10), over-co-credited (*n* = 6.5), and under-credited (*n* = 8.5). Examples of over-crediting include insufficient credit given to Pierre Curie and Gustave Bémont for their contributions on polonium and/or radium^21,22^ and to André-Louis Debierne for his assistance on the isolation of radium metal.^19^ However, half of the cases in which Marie Curie is over-credited occur when texts state that the curie and curium were named in her honor. The curie, in fact, was named to honor Pierre Curie,^39^ and curium was named for both Marie and Pierre Curie.^40^ The curie/curium over-crediting, however, has another dimension. It implies a significant degree of peer support for Marie Curie, which was not always the case.^41^ This characterization can camouflage the contextual realities and, consequently, under-credit Marie Curie for the obstacles she overcame.

Several instances of over-co-crediting were observed in the Marie Curie transcripts. For example, the statement “In 1903 she and her French husband, Pierre Curie, were awarded the Nobel Prize in Physics for their work on radioactivity”^18^ does not acknowledge that this award was also shared with Henri Becquerel and, thereby, over-co-credits both Marie and Pierre Curie.

Marie Curie is also explicitly under-credited (*n* = 8.5). Examples include statements that she was Becquerel’s student (e.g., “one of Becquerel’s students”,^18^ “began her doctoral work with Henri Becquerel”),^19^ which imply she may not have been the driving force behind the work. Marie Curie was not Becquerel’s student. Her doctoral thesis lists Gabriel Lippmann as the president of the thesis jury,^42^ which indicates he served as the thesis director. Henri Becquerel is not even listed in Marie Curie’s dissertation as a member of the science faculty.^42^

Some statements were not explicitly related to over/under-crediting but impact the reader’s perception. For example, one text states, “Marie Curie herself succumbed to aplastic anemia caused by years of research with radioactive materials.”^21^ Data from her 1995 exhumation indicates that Marie Curie’s exposure to X-rays during the First World War was the more likely culprit in her illness.^43,44^ No textbooks mention Marie Curie’s courage, dedication, and life-saving work on the battlefields during the war. Thus, not only is the information misleading but her heroism is ignored while simultaneously perpetuating the trope that female scientists must sacrifice their lives to achieve success.

In summary, attribution mistakes occur less frequently for Linus Pauling, but all attribution errors over-credit/co-credit Pauling and thereby elevate his status. The attribution errors for Marie Curie are more varied. She is over-credited/co-credited, but many statements implicitly under-credit her accomplishments and/or may be attenuated by doubt raisers. Marie Curie is also explicitly under-credited, and such instances can diminish the reader’s perception of her status.

Over-crediting and under-crediting are both inherently problematic and affect male and female scientists. The under-credited scientists become invisible in the textbooks, and the impression formulated by the reader is based solely on what is said about the featured scientists (in this case, Linus Pauling and Marie Curie). The erasure of other contributing scientists obscures the importance of collaboration and interdependence within the scientific enterprise. In addition, the recipient of over-crediting can be elevated to a superhuman status without appropriate context,^45^ which may limit their capacity to serve as a relatable role model.

Textbook inaccuracies were also observed that extended beyond the attribution of credit. There were instances of incorrect indexing, improperly transcribed direct quotes, incorrect data, misrepresentations of the content of research papers, and a mislabeled photo of “Marie Sklodowska Curie”. The number and variety of inaccuracies indicate a spectrum of quality control issues in the production of chemistry textbooks. Thus, the inaccuracies are likely to have a range of root causes and potential outcomes. This topic is beyond the scope of this manuscript, and further research is warranted.

## Conclusions

Textbook descriptions of Marie Curie and Linus Pauling differ in length, language, and content. Less text is written about Marie Curie, and the language includes more sex-linked terms, subordinate language, and communal/familial connections. Descriptions of Marie Curie’s work include more grindstone language, and her accomplishments are often under-credited and/or attenuated by doubt raisers. Descriptions of Linus Pauling are longer and include more agentic references, less communal/familial terms, and a lack of subordinate language. His accomplishments are amplified by superlatives, repetition of standout words, over-crediting, and less doubt raisers.

The language and inaccuracies in textbooks construct caricature images of scientists that reinforce stereotypes, lead to the erasure of realistic role models, and may deter entry and retention in the discipline. Linus Pauling’s textbook caricature, for example, is a genius who makes discovery after discovery, excels in many areas of science, does not get his hands dirty, saves the world, and has no apparent flaws. This idyllic portrayal belies biographical descriptions of Pauling as egotistical, narcissistic, excessively litigious, claiming more credit than he is due, and driven by being in the limelight.^46^ In addition, the characterization of truly exceptional aptitude and extreme independence as central to scientific success for males is problematic. It may deter talented students who are modest or lack confidence, amplify bravado, or make it difficult to admit mistakes. Furthermore, the “solo brilliant scientist” imagery conceals the importance of skills such as teamwork and communication that are critical in the modern scientific workplace.

In some instances, the over-crediting of Pauling is at the expense of scientists from marginalized and underrepresented groups, which robs students of the opportunity to learn about scientists with diverse backgrounds. For example, Herman Branson and Robert Corey were critical to the early discoveries of protein structure, but Pauling is given sole credit in the textbooks. Branson was an acclaimed African American physicist and college president, while Corey, partially paralyzed from polio, was a preeminent crystallographer and a namesake of CPK models.^47,48^ Ignoring Branson and Corey’s contributions is a missed opportunity to highlight their stories and teach students that the most impactful scientific work comes from diverse research groups.^49^

The elevation of an individual scientist to the detriment of others is not new. In 1968, Merton named this phenomenon the “Matthew Effect”,^50^ which he defined as the “enhancement of the position of already eminent scientists who are given disproportionate credit in cases of collaboration or of independent multiple discoveries”. Merton noted how the misattribution of credit leads to “cumulative advantage” and, when “transformed into an idol of authority”, it “curbs the advancement of knowledge”. In essence, this practice elevates a select number of scientists and occurs to the detriment of the discipline and the public.

Marie Curie’s textbook caricature is a tireless workhorse, willing to endure harsh conditions, shoveling pitchblende day in and day out, guided by the men around her, and ultimately achieving the coveted prize of being recognized by the men in the discipline. The notion that females need to make extreme sacrifices to achieve success in the workplace sets the stage for them to be exploited. This mentality can permit serial predators to thrive in the STEM fields and may exacerbate the exodus of talented individuals.^51^

Marie Curie is also under-credited in the textbooks, and the phenomenon of systemically ignoring, minimizing, or denying credit to female scientists was described and named the “Matilda Effect” by Rossiter.^15^ Despite calling attention to this problem 30 years ago, the practice continues.

The texts also suggest that Marie Curie was showered with honors (such as naming the curie and curium after her), which likely exaggerates the actual degree of peer and public support.^41^ The implication of support can obfuscate problematic attitudes and behaviors and, in turn, allow them to persist. It also can perpetuate the illusion of scientists as objective because if bias (conscious or unconscious) is recognized, then a lack of objective evaluation must be acknowledged.

On a final note, it is worthwhile to consider the possibilities if we adopted fundamentally different textbooks. For example, what if STEM textbooks embraced inclusivity, work-life balance, ethics, and social justice? What impact might this have? Could this repair the breakdown of trust between scientists and the public? Could diverse STEM teams find ways to address climate change and healthcare disparities? While changing textbooks may not be the only step necessary to address complex global issues, it is one step. And, if the barrier to make a change to a textbook is too high, what does this say about the capacity of the STEM workforce to innovate and adapt? Will we be able to make the changes necessary to allow future generations to prosper?

## Supporting Information Available

Elaboration of scientist selection process, index accuracy analysis, details of counting methodology, data tables, additional examples of transcript words and phrases used for quantification, method details for accuracy analysis, commentary on Nobel prize biases, connection to student evaluations of teaching, comparison between textbook data and performance evaluations, and analysis of textbook photos (PDF, DOCX)
