# Supplementary material for: Differences in the Portrayal of Female and Male Scientists in College Chemistry Textbooks
Source: J Chem Educ. 2025 Apr 9;102(5):1912–7. doi: 10.1021/acs.jchemed.4c01365 (PMC12080112; doi:10.1021/acs.jchemed.4c01365)
Supplement: Supplementary file 2 — ed4c01365_si_002.docx [file ed4c01365_si_002.docx]

Supporting Information

Differences in the Portrayal of Female and Male Scientists in College Chemistry Textbooks

Peyton T. Fair ^1,2^, Melanie R. Nilsson ^3*^

**AUTHOR AFFILIATIONS:**

^1^ Department of Biology, McDaniel College, Westminster, MD 21157, USA.

^2^ Current address: Lewis Katz School of Medicine at Temple University / St. Luke’s University Health Network, Bethlehem, PA 18015, USA.

^3^ Department of Chemistry, McDaniel College, Westminster, MD 21157, USA.

*****Corresponding author: Melanie R. Nilsson, Email: [mnilsson@mcdaniel.edu](mailto:mnilsson@mcdaniel.edu); Ph: 410-857-2799.

**MATERIALS AND METHODS:**

**Selection of Scientists.** Marie Curie was chosen for this study because she is the most frequently indexed female scientist in General Chemistry textbooks. In the 10 textbooks, a total of 12 females are indexed ^1^. Of the twelve, only five appear in more than one text. These five include the following individuals (the number of texts appears in parenthesis): Marie Sklodowska Curie (8), Lise Meitner (5), Rosalind Franklin (4), Irène Joliot-Curie (3), and Maud Menten (2).

Marie Curie is the only person to win Nobel prizes in two different fields of science (chemistry and physics), making her status unparalleled. Only four other individuals have won two Nobel prizes of any type (Linus Pauling, John Bardeen, Frederick Sanger, and Barry Sharpless). Since Bardeen, Sanger, and Sharpless are not mentioned in General Chemistry textbooks, Linus Pauling was chosen for comparison. Linus Pauling won one Nobel prize in chemistry and the other in peace. He is featured in all 10 General Chemistry textbooks.

Marie Curie and Linus Pauling both attained Ph.D. degrees, were faculty members, contributed to wartime research efforts (Cure in WW I and Pauling in WW II), extended their research into the medical field, and received two Nobel prizes. On a personal level, they also shared similarities. They were both white, heterosexually married, and had children. These similarities enable a reasonable comparison but also limit the scope of conclusions that can be drawn. For example, the textbook descriptions of Curie and Pauling may not encompass the experience of scientists from different demographic populations (e.g. People of Color, LGBTQ+).

Marie Curie and Linus Pauling differ in ways that extend beyond being perceived as male and female. Marie Curie was born in Poland, moved to France to obtain her advanced degrees, and primarily conducted research in France. Linus Pauling was born in the US, received his education in the US, and primarily conducted his research in the US. Since the textbooks in this study are US textbooks, the different nationalities of the scientists may contribute to differences observed in the textbook language and descriptions. The two scientists also lived and worked during different time periods, Marie Curie (1867-1934) and Linus Pauling (1901-1994), which may contribute to how they are depicted.

**Identification of Passages and Index Accuracy.** All indexed mentions of Pauling were ascribed to Linus Pauling. For Marie Curie, only references to Marie Curie or Madame Curie were included since many texts also mention Pierre or Irène Curie. However, using this criterion, only one sentence was excluded. This sentence was on an unindexed page that mentioned “Curie” with no other modifiers ^2^. Since the statement could not be unambiguously ascribed to Marie Curie, the sentence was not included. Indexed entries for curie, the unit of measure, were not included.

For both Marie Curie and Linus Pauling, the actual number of unique pages listed in the index was recorded as well as the number of falsely indexed and unindexed pages. A false index was operationally defined as a page in the book that was indexed under the scientist yet did not mention the scientist on that page. An unindexed entry was defined as a page that mentioned the scientist’s name but was not included in the index. Instances of false indexing and unindexed pages were typically not correlated; only one text ^3^ contained both types of errors.

The average number of indexed pages per book for Marie Curie (average=2, median=1.5) is lower than for Linus Pauling (average=2.4, median=3); this is driven, in part, by the lack of an indexed entry for Marie Curie in two of the texts ^4^. The difference, however, is more pronounced than these numbers suggest because, in two of the eight textbooks in which Marie Curie is indexed, she is not indexed as an individual. In these cases, Marie Curie is co-indexed with Pierre Curie ^3, 5^. Since the index entries could not be decoupled, the number of indexed pages ascribed to Marie Curie is less than the reported average and median. Furthermore, one text contains an indexed page for Linus Pauling that refers to an online extra chapter that was not included in the above count ^6^.

The total number of falsely indexed pages across the 10 books is higher for Marie Curie (n=2) than for Linus Pauling (n=0) and encompasses 25% of texts with indexed entries for Marie Curie and 0% for Linus Pauling. Therefore, an individual seeking information about Marie Curie may not be able to easily locate information about her. This is a particular concern since so few women are mentioned in the textbooks. The total number of unindexed pages across the 10 books is lower for Marie Curie (n=5) than for Linus Pauling (n=10). Both scientists, therefore, are mentioned more frequently than the index alone would indicate, but the unindexed frequency is twice as high for Pauling.

**Agentic and Communal Proxies.** Name counts for Linus Pauling include every use of the name Pauling or Linus Pauling. Name counts for Marie Curie include those that mention Marie Curie or Madame Curie. References to “the Curies” as a couple were multiplied by one half to account for Marie Curie being only one half of the pair. Other people were quantified by counting every name that did not refer to the scientist. “Curies” was recorded as one-half in the “other” category since Pierre Curie is included in this reference. Curie, the unit of measure, was not counted as a name.

**Standout, Grindstone, and Doubt Raiser Language.**

Coders analyzed the transcripts for standout, grindstone, and doubt raiser words or phrases. Published lists of words and word roots for specific categories were used as a guide ^7, 8^, but did not limit the search. For each category, words or phrases were included if they referred to the scientist of interest, either alone or with others.

Coders did not sub-classify items in each category. For example, doubt raisers were not sub-classified into whether they were hedges, unexplained comments, faint praise, etc. Similarly, standout words were not subclassified into superlatives, phrases that describe exceptionality, etc.

In this study, there were not a unique number of items to evaluate. The transcripts could be interpreted differently by each coder in terms of the number of applicable words/phrases. Therefore, the extent of agreement between the coders was assessed by calculating the number of identical ratings of the two coders divided by the average number of ratings for the two coders and then multiplying by 100%. This approach allows for variability in the number of ratings reported by each coder.

The extent of agreement for each variable was determined to be: standout = 95%, grindstone = 97%, and doubt raiser = 98%. The overall agreement was determined to be 96% by using the total number of identical ratings across all categories (n=116) and the average number of ratings for the two coders (n=120.5).

**Nationality.** Quantification of nationality included adjectives used to describe the scientists (e.g., Polish, American) or references to their birthplace. Locations of where the scientists studied or worked were not included.

**Accuracy of Descriptions.** The transcripts were examined for accuracy. Textbook figures and tables that were referenced within the transcripts were included in the analysis. The total number of inaccuracies in a figure or table was not quantified, but each reference from the transcript to an inaccurate figure or table was counted as an inaccuracy. For consistency, in the interpretation of the transcripts, all derivatives of the word “develop” were interpreted as originate (as opposed to evolve or advance).

Each element of text was compared to appropriate sources and sorted into color-coded categories as follows: Green = accurate, Pale Green = accurate, but can also be interpreted in a way that would be inaccurate, Orange = could not be explicitly determined to be accurate or inaccurate, Dark Orange = could not be explicitly determined to be accurate or inaccurate, but evidence suggests the claim is more likely to be inaccurate, Red = inaccurate.

Green statements commonly included birth and death years, references to Nobel prizes won, statements regarding scientific findings, etc. An example of a statement categorized as Pale Green is: “In the 1930s, Linus Pauling and his coworkers conducted a systemic investigation of protein structure” ^9^. The scientific papers published on this topic indicate the work began in the 1930s, but Pauling and his coworkers also conducted significant work after this decade ^10^. Thus, the statement is accurate but could be misinterpreted to mean that all the work was completed in the 1930s. Both Green and Pale Green statements were considered accurate in the analysis, but the nuanced differences were noted during data collection.

Orange statements are those in which no clear determination could be made. One such statement was: “Newspaper articles published in the 1920s made clear that Marie Curie was deeply troubled by the tragedy of the Radium Girls…”^11^. We searched newspaper databases in both English and French and found no references to corroborate this statement. We also consulted experts in both the US and France, and neither had encountered any such articles. No citation was provided in the textbook for this statement, so an original source could not be consulted to verify the accuracy. The brevity of the statement made it impossible to disprove since an article could have been written in a language other than English/French or may not have been indexed in the searched databases. Thus, it was categorized as Orange to reflect a statement that we were unable to determine if it was accurate or inaccurate.

Dark Orange statements were those that could not be explicitly proven or disproven, but the evidence suggests the claim is more likely to be inaccurate. For example, one textbook states that Pauling developed the “most widely used” electronegativity scale^12^. No citation or data to substantiate the claim was provided in the textbook. Our literature searches revealed no study to indicate this electronegativity scale to be the one that is used most widely. The term “used” is unclear, but we interpret the statement to indicate the scale being “used” by scientists as a measure of electronegativity. A search of the scientific literature revealed articles in which Pauling’s scale was reported to be inaccurate and inferior to other scales^13, 14^. Since we found no explicit data to substantiate the claim and primary literature articles indicate the statement is inaccurate, this statement was categorized as Dark Orange.

Red statements are those determined to be inaccurate. For example, one textbook labeled an image as “Marie Sklodowska Curie” ^6^, but it is not actually a photo of Marie Sklodowska Curie. See the Results section of this SI for the detailed analysis conducted in determining this statement to be inaccurate.

In quantifying the data, Dark Orange and Red statements were classified as inaccurate. Since this study is focused on the portrayal of the scientists, inaccuracies related to the attribution of credit were sub-categorized as over-credit, over-co-credit, or under-credit. Linus Pauling was over-credited (n=24), and over-co-credited (n=1). Marie Curie was over-credited (n=10), over-co-credited (n=6.5), and under-credited (n=8.5). In one instance, Marie Curie was simultaneously over-co-credited and under-credited, so a 0.5 count was allotted to each category. Since the transcript length was different for the two scientists, frequency is reported in the manuscript to provide a more useful comparison.

**RESULTS AND DISCUSSION:**

**Data Summary.**

Raw and processed quantitative data for parameters measured in this study are summarized in Tables 1-3.

Table 1: Sex-linked terms, Subordinating Language, Agentic and Communal Proxies, Familial Terms, and Nationality

| **Parameter** | **Marie Curie** | | **Linus Pauling** | |
| --- | --- | --- | --- | --- |
|  | **n** | **% words** | **n** | **% words** |
| Sex-linked Terms | 55 | 3.9 | 27 | 1.5 |
| Subordinating Language | 5 | 0.4 | 0 | 0.0 |
| Agentic Proxy | 44.5 | 3.2 | 75 | 4.2 |
| Communal Proxy | 39.5 | 2.8 | 11 | 0.6 |
| Familial Terms | 19 | 1.4 | 1 | 0.1 |
| Nationality | 13 | 0.9 | 7 | 0.4 |

The Marie Curie transcript contains 1399 words and the Linus Pauling transcript contains 1804 words. The % words column reflects the parameter occurrence as a percentage of the total word count.

Table 2: Standout, Grindstone, and Doubt Raiser Language

| **Parameter** | **Marie Curie** | | | **Linus Pauling** | | |
| --- | --- | --- | --- | --- | --- | --- |
|  | **n_total_** | **n_shared_** | **1/frequency** | **n_total_** | **n_shared_** | **1/frequency** |
| Standout | 28 | 13 | 4.4 | 28 | 1 | 4.4 |
| Grindstone | 27.5 | 13 | 4.5 | 8.5 | 1 | 15.3 |
| Doubt Raiser | 22 | 6 | 4.9 | 6.5 | 0 | 18.8 |

N_total_ is the total number of descriptors of that type within the transcript. N_shared_ is the number of descriptors that modify the scientist of interest as well as another person. The parameter 1/frequency is the inverse of (n_unshared_ + 0.5 x n_shared_)/number of lines of text. The Marie Curie transcript contains 94 lines and Linus Pauling contains 122 lines.

Table 3: Types of Attribution Inaccuracies

| **Type of attribution inaccuracy** | **Marie Curie** | | **Linus Pauling** | |
| --- | --- | --- | --- | --- |
|  | **n** | **%** | **n** | **%** |
| Over-credit | 10 | 40 | 24 | 96 |
| Over-co-credit | 6.5 | 26 | 1 | 4 |
| Under-credit | 8.5 | 34 | 0 | 0 |

The % column expresses the specific type of attribution inaccuracy as a percentage of the total number of attribution inaccuracies for the given scientist.

**Subordinating Language**. On two occasions the term “Madame” was used ^3,6^; this word is the French equivalent of “Mrs.” Other uses of subordinating language include one reference to Marie Curie as a student, another as “One of Becquerel’s students” ^9^, and that she worked on radioactivity “At Becquerel’s suggestion” ^12^. The latter statements not only have a subordinating effect but are also inaccurate.

**Agentic and Communal Proxies.** The other individuals mentioned with Marie Curie include 21.5 mentions of Pierre Curie (Pierre + one-half “Curies”), nine of Henri Becquerel, four of Iréne Joliot-Curie, three of Frederic Joliot-Curie, and two mentions of Ernest Rutherford. With Linus Pauling, there was one mention each of Walter Heitler, Fritz London, John Slater, Francis Crick; two references to Allred and Rochow; and three mentions of Robert Mulliken.

The familial terms used in passages about Marie Curie include husband (n=11), daughter (n=4), and one each of son-in-law, married, wife, and parent. For Linus Pauling, there was a single use of the word “wife”.

**Standout Descriptors.**

*Nobel prize biases.* Nobel prizes are the most common standout descriptor for both Curie (66%) and Pauling (38%). The awarding of the Nobel prizes, however, is imperfect ^15^ and skewed by gender ^16^. The textbooks lack any acknowledgment of the potential biases in the award-granting process and this may perpetuate the myth of science as a meritocracy. Nobel prizes could be used as a springboard to discuss the potential for cognitive errors and bias in science ^17^, topics that would elevate the critical thinking skills of students.

Marie Curie’s standout descriptors primarily relate to Nobel prizes (66%) or the naming of the curie and curium (14%). Additional standout descriptors include: “pioneering work on radioactivity (a term she introduced)” ^12^, “great contribution to science” ^9^, “first to coin the term ‘radioactivity’” ^5^, “launch a new branch of chemistry” ^11^, “significant development in nuclear chemistry” ^3^, and “first new radioactive element” ^6^.

There are also four mentions that Marie Curie was the first woman to win a Nobel prize or the only woman to win two, which were counted as standout descriptors. This language, however, implies she is the best among female scientists, not necessarily the best among all scientists. In addition, others have noted that such “firsts” may say more about the biases within the prize committee than about the person receiving the award ^18^.

Two standout phrases about Marie Curie contained comingled terms that were perceived to negatively modify the impact. In “*helping* launch a new branch of chemistry” ^11^, the word “helping” can reflect a subsidiary role and thereby attenuate the praise. Also, in “*Despite* her great contribution to science” ^9^, the use of “Despite” was perceived to have a diminishing effect.

Linus Pauling’s standout descriptors frequently mention Nobel prizes (38%), but a variety of other forms of praise are included, such as “first and most widely used“ ^12^, “most influential chemist of the twentieth century“ ^9^, “remarkably broad“ ^9^, “great triumph“ ^9^, “more widely used“ ^25^, “one of the most famous chemists of the twentieth century“ ^5^, “foundational to our current understanding of chemistry“ ^5^, “many important contributions“ ^5^, “genius“ ^11^, “so useful“ ^3^, “seminal“ ^3^, and “one of the founders“ ^3^.

One standout phrase related to Pauling’s electronegativity values states “Because they are so useful, Pauling’s values have been continually refined since Pauling’s 1932 publication” ^3^. The phrase “so useful” was noted as a standout descriptor, but the mention of “continually refined” indicates the values are not that useful and thereby attenuate the impact.

*Connection to student evaluations of teaching:* The specific standout words “genius” and “brilliant” were previously reported to be more common in student evaluations of male versus female chemistry teachers ^19^. We found that “brilliant” was not used to describe Curie nor Pauling. “Genius” was never used to describe Marie Curie but appeared in one description of Linus Pauling ^11^. While this is consistent with the trend observed by Storage et al., the limited data prohibits any definitive conclusions.

**Grindstone Language.**

Marie Curie grindstone terms frequently include derivatives of the words “isolate”, “separate”, or “extract” (56%) and the word “work” (29%). Other grindstone descriptors include “It took them four more years” ^25^, “Finally” ^25^, “years of research” ^11^, and “only about one gram of radium-226 was present in several tons of uranium ore processed by Marie Curie” ^6^. Repetition of grindstone words within a single phrase were observed (e.g., “separated and purified” ^11^, “painstakingly separated” ^3^), but were only counted as one instance of grindstone language. The phrase “pioneering work” ^11-12^ was used on two occasions and, while the word “work” was counted as grindstone, the impact is attenuated by the adjective “pioneering”.

The most common grindstone term used in reference to Linus Pauling was “work” (71%). Other descriptors include “conducted a systemic investigation” ^9^, “efforts” ^11^, and “earned” ^3^. There was no repetition of grindstone language within a single phrase, but several instances (47%) were attenuated by standout adjectives (e.g., “work was a great triumph” ^9^, “work was also pivotal” ^5^, “seminal work” ^3^, “genius and efforts” ^11^).

**Doubt Raisers.**

Doubt raisers in the Marie Curie transcripts relate to her scientific work and to her as a person. Some sentences may raise doubt in more than one way (e.g. hedging language, faint praise, irrelevancies, unexplained/potentially negative comments) but were only counted as one instance of a doubt raiser.

Marie Curie is described as “a young polish doctoral student” ^2^, and the word “young” can imply inexperience. Several statements also indicate she was working under the direction of Becquerel (e.g., “At Becquerel’s suggestion” ^12^, “One of Becquerel’s students” ^9^) which raise doubt as to Marie Curie’s contributions to the work. Other instances of subordinating language, such as the use of “Madame”, were not counted as doubt raisers.

Statements regarding Marie Curie’s cause of death are irrelevant and may have negative connotations. It is stated that she “succumbed to aplastic anemia caused by years of research” ^11^ and “died from radiation exposure” ^11^. It is also noted that radiation exposure was the cause of death of “her daughter Irène Joliot-Curie, who continued the research program started by her parents” ^11^. The cause of death is not mentioned for Linus Pauling, nor any other males within any of the transcripts. The descriptions of Marie Curie’s death also suggest a lack of laboratory safety, which could imply carelessness. Statements like “Marie Curie was deeply troubled by the tragedy of the Radium Girls” ^11^ may further perpetuate the idea that Marie Curie was, in some way, culpable.

Some co-mentions of other scientists were counted as doubt raisers, such as the statement “Her daughter Irene, and son-in-law Frederic Joliot-Curie, shared the Nobel Prize in Chemistry in 1935” ^9^. Most references to Irène Joliot-Curie primarily emphasize familial relationships rather than illuminate accomplishments or scientific work and, thereby, do not seem relevant.

Some additional doubt raiser examples include (doubt raisers highlighted in italics): “working in an *old shed*” ^3^, “*Nevertheless*, she was successful” ^6^, “*It is a credit* to her skills as a chemist” ^3^, “detect *but not isolate*” ^6^, “*Despite her* great contribution to science” ^9^. Other examples highlight that the curie unit is obsolete but provide no explanation (e.g., “*An older* radioactivity unit is the curie” ^11^).

All doubt raisers in the Linus Pauling transcripts relate to his scientific work. The majority (62%) are caveats to Pauling’s original electronegativity scale (e.g., “Other electronegativity scales have been developed with slightly different values from those proposed by Pauling” ^11^, “A variety of methods for quantifying electronegativity values have been developed since Pauling’s initial work” ^3^). Other doubt raisers include cases in which nothing explicitly negative is stated but there is an implication that something might be unclear: “In *the original* Pauling theory of hybridization” ^3^ and “*In general*, the conditions necessary to form noble gas compounds are as Pauling predicted.” ^6^

**Degree of Others Co-Mentioned in Descriptors.**

The frequency with which other people are co-mentioned with the descriptors (standout, grindstone, doubt raiser) is higher for Marie Curie in comparison to Linus Pauling (Table 4). This is consistent with the communal proxy data in which other people were determined to be co-mentioned almost five times more frequently with Marie Curie in comparison to Linus Pauling. It is also interesting that accolades and work are more frequently “shared” within the descriptions in comparison to doubt. Further study will be needed to determine if there is any significance to this observation.

Table 4: Percentage of Descriptors Co-Modifying Others

| **Descriptor** | **Marie Curie** | **Linus Pauling** |
| --- | --- | --- |
| Standout | 46% | 4% |
| Grindstone | 47% | 12% |
| Doubt Raiser | 27% | 0% |

**Comparison of Textbook Analysis and Performance Evaluations.**

*Length.* Prior studies on recommendation letters demonstrate that the gender of the writer can impact length ^20, 21^. Therefore, the textbook author names and pronouns were examined, and the perceived gender distribution of textbook authors was determined to be 83% male and 17% female. This is comparable to the letter writers reported by Trix & Psenka (85% male, 12% female, 3% unknown) and Turrentine et al. (86% male, 14% female) ^7, 20^. Trix & Psenka and Turrentine et al. report higher average word counts in recommendation letters written for male applicants compared to female applicants, which mirrors our findings of more text written for Pauling in comparison to Curie.

*Sex-linked terms.* In letters of recommendation, Trix & Psenka found sex-linked terms are used twice as frequently for female versus male applicants ^7^, which parallels our findings. The higher mention of sex-linked terms with respect to women may indicate that the writers see male as the “norm” and mention a female’s sex to highlight a deviation from the “norm”.

*Subordinating Language.* Trix & Psenka report a greater use of distinctive titles to describe male applicants in letters of recommendation, and Files et al. found that male hosts used formal titles more frequently when introducing a male speaker versus a female speaker ^7, 22^. The lower degree of subordinating language for Linus Pauling in comparison to Marie Curie is consistent with these studies.

*Agentic and Communal Proxies.* Madera et al. found more agentic terms in letters written for men and more communal terms in letters written for women ^23^, which aligns with our proxy data. Turrentine et al. found the applicant’s name to be more common in letters of recommendation written for male applicants, similar to the more frequent use of Linus Pauling’s name. They did not find any gender difference in specific agentic or communal words but suggest this may be due to the high value placed on teamwork for the job in their study (surgical residents) ^20^. Trix & Psenka found more references to “personal life” for women ^7^, which is consistent with the higher number of familial terms in the textbook passages about Marie Curie.

*Grindstone.* Turrentine et al. observed more grindstone words and references to work ethic in recommendation letters written for female applicants ^20^, which parallels the textbook data.

*Standout.* Trix & Psenka found a higher average number of standout adjectives with a greater degree of repetition in letters written for male applicants^7^. Turrentine et al. observed a higher prevalence of standout words in letters written for male applicants and “positive statements supporting female applicants seemed more likely to include a qualifier” that attenuated the praise ^20^.

The Marie Curie and Linus Pauling transcripts have the same frequency of standout descriptors (one every 4.4 lines of text), which differs from the higher average number for male applicants reported by Trix & Psenka and Turrentine et al. However, the Pauling transcripts contained a higher number of superlatives and repetition of standout words, which is consistent with Trix & Psenka. There were also more qualifiers in reference to Marie Curie’s accomplishments, which aligns with observations by Turrentine et al.

*Doubt Raisers.* Trix & Psenka found twice as many recommendation letters written for female applicants contain a doubt raiser in comparison to letters written for male applicants. In addition, of the letters that contain doubt raises, there were a higher number of them per letter for female applicants ^7^. Other studies have also found more doubt raises in letters written for female candidates ^20, 24^. The degree to which textbooks embed doubt raisers in the descriptions of Marie Curie correlates with the observations of high numbers in recommendation letters for female candidates. Madera et al. notes that both male and female letter writers use more doubt raisers for female candidates ^24^.

**Accuracy.**

*Textbook Photos of Marie Curie and Linus Pauling.* The total number of images of Marie Curie in the textbooks (n=4) is higher in comparison to Linus Pauling (n=3). A fifth image is labeled “Marie Sklodowska Curie” in one textbook ^6^, but is not actually a photo of Marie Curie. Therefore, it is not included in the image count.

The incorrect photo was initially identified by a lack of visual correlation to other photos of Marie Curie. It was subsequently used as the input for a reverse image search on the internet using Bing images and the PimEyes image search software. Both Bing and PimEyes identified this photo as Irène Joliot-Curie. The textbook with the mislabeled photo had a separate image with a caption indicating it was Irène Joliot-Curie. The Irène Joliot-Curie photo was determined to be correct, which indicates this was not simply a mix-up in the placement of the two images. The textbook containing this photo error ^6^ includes a total of 7 female scientists and 167 male scientists in the index ^1^. During a prior study ^1^, we incidentally noticed this textbook contained two other anomalies with regard to female scientists. Ida Tacke Noddack’s name is misspelled in both the text and index, and Laila Suidan’s name is abbreviated L. Suidan throughout. The textbook is in its 11^th^ edition, which suggests there may be systemic issues in the revision and review processes.

All images of Marie Curie are in black and white. One photo of Marie Curie depicts her reading a paper with eyes so downcast as to not be visible ^2^, while another shows Pierre Curie standing over her while she works in a lab wearing a full skirt that occupies almost 25% of the image ^25^. The remaining two photos are plain headshots ^3, 12^. The images of Linus Pauling include two color photos and one in black and white. Two photos show him in a suit next to models of atomic structures and, in both, he does not directly look at the camera but glances to the side with a slight smile ^3, 5^. In the third image, he looks pensively at the camera with head in hand ^3^.

Although there are more photos of Marie Curie than Linus Pauling, the portrayals of Marie Curie are less likely to positively resonate with modern students. The three headshots of Marie Curie are black and white with no indication of her scientific background, while the headshots of Linus Pauling include two in color and two with molecular models that reference his scientific expertise. The one photo of Marie Curie in the lab shows Pierre Curie standing over her as if he is overseeing the work, which may falsely imply that she was subordinate to him. Furthermore, neither are wearing any type of safety protection in the image (e.g., lab coat, goggles, etc.) and this may be interpreted as carelessness or unprofessionalism to a modern student.

**CONCLUSIONS:**

*Another opportunity to feature a diverse scientist:* Harvey Itano, who made key contributions to understanding sickle-cell anemia (for which Pauling is given credit in the textbooks), could also be featured. Itano’s research was disrupted by interment in a US prison camp due to his Japanese heritage ^26^. His story could provide an interesting opportunity to discuss the negative impact of American xenophobia on scientific progress.

**SUPPORTING INFORMATION REFERENCES:**

(1) Becker, M. L.; Nilsson, M. R. College Chemistry Textbooks Fail on Gender Representation. *Journal of Chemical Education* **2021**, *98* (4), 1146-1151.

(2) Atkins, P. W.; Jones, L.; Laverman, L. *Chemical principles : the quest for insight*, 7^th^ ed.; W.H. Freeman, 2016.

(3) Kotz, J. C.; Treichel, P.; Townsend, J. R.; Treichel, D. A. *Chemistry & chemical reactivity*, 10^th^ ed.; Cengage, 2019.

(4) Robinson, J. K.; McMurry, J. E.; Fay, R. C. *Chemistry*, 8^th^ ed.; Pearson, 2019. Zumdahl, S. S.; Zumdahl, S. A.; DeCoste, D. J. *Chemistry*, 10^th^ ed.; Cengage, 2018.

(5) Flowers, P.; Theopold, K.; Langley, R.; Robinson, W. R. *Chemistry 2e*; OpenStax, 2019.

(6) Petrucci, R. H.; Herring, F. G.; Madura, J.; Bissonnette, C. *General chemistry : principles and modern applications*, 11^th^ ed.; Pearson, 2017.

(7) Trix, F.; Psenka, C. Exploring the Color of Glass: Letters of Recommendation for Female and Male Medical Faculty. *Discourse & Society* **2003**, *14* (2), 191-220. DOI: 10.1177/0957926503014002277.

(8) Schmader, T.; Whitehead, J.; Wysocki, V. H. A Linguistic Comparison of Letters of Recommendation for Male and Female Chemistry and Biochemistry Job Applicants. *Sex roles* **2007**, *57* (7-8), 509-514. DOI: 10.1007/s11199-007-9291-4.

(9) Chang, R.; Overby, J. *Chemistry*, 13^th^ ed.; McGraw-Hill Education, 2019.

(10) Pauling, L.; Corey, R. B.; Branson, H. R. The structure of proteins; two hydrogen-bonded helical configurations of the polypeptide chain. *Proc Natl Acad Sci U S A* **1951**, *37* (4), 205-211. DOI: 10.1073/pnas.37.4.205 From NLM. Pauling, L.; Corey, R. B. The pleated sheet, a new layer configuration of polypeptide chains. *Proc Natl Acad Sci U S A* **1951**, *37* (5), 251-256. DOI: 10.1073/pnas.37.5.251 From NLM.

(11) Gilbert, T. R.; Kirss, R. V.; Foster, N.; Bretz, S. L.; Davies, G. *Chemistry*, 5^th^ ed.; W.W. Norton, 2018.

(12) Brown, T. L.; LeMay, H. E.; Bursten, B. E.; Murphy, C. J.; Woodward, P. M.; Stoltzfus, M. W. *Chemistry : the central science*, 14^th^ ed.; Pearson, 2018.

(13) Sproul, G. D. Evaluation of Electronegativity Scales. *ACS Omega* **2020**, *5* (20), 11585-11594. DOI: 10.1021/acsomega.0c00831.

(14) Murphy, L. R.; Meek, T. L.; Allred, A. L.; Allen, L. C. Evaluation and Test of Pauling's Electronegativity Scale. *The Journal of Physical Chemistry A* **2000**, *104* (24), 5867-5871. DOI: 10.1021/jp000288e.

(15) Merton, R. K. The Matthew effect in science. The reward and communication systems of science are considered. *Science* **1968**, *159* (3810), 56-63. Friedman, R. M. *The Politics of Excellence: Behind the Nobel Prize in Science*; W.H. Freeman, 2001.

(16) Modgil, S.; Gill, R.; Lakshmi Sharma, V.; Velassery, S.; Anand, A. Nobel Nominations in Science: Constraints of the Fairer Sex. *Ann Neurosci* **2018**, *25* (2), 63-78. DOI: 10.1159/000481906 From NLM.

(17) Nuzzo, R. How scientists fool themselves – and how they can stop. *Nature* **2015**, *526* (7572), 182-185. DOI: 10.1038/526182a.

(18) Brainard, C. ‘The Finkbeiner Test’ Seven rules to avoid gratuitous gender profiles of female scientists. *Columbia Journalism Review* **2013**. (acccessed January 19, 2023).

(19) Storage, D.; Horne, Z.; Cimpian, A.; Leslie, S. J. The Frequency of "Brilliant" and "Genius" in Teaching Evaluations Predicts the Representation of Women and African Americans across Fields. *PLoS One* **2016**, *11* (3), e0150194. DOI: 10.1371/journal.pone.0150194.

(20) Turrentine, F. E.; Dreisbach, C. N.; St Ivany, A. R.; Hanks, J. B.; Schroen, A. T. Influence of Gender on Surgical Residency Applicants' Recommendation Letters. *J Am Coll Surg* **2019**, *228* (4), 356-365.e353. DOI: 10.1016/j.jamcollsurg.2018.12.020.

(21) Watson, C. Sex-Linked Differences in Letters of Recommendation. *Women and Language* **1987**, *10* (2), 26-28.

(22) Files, J. A.; Mayer, A. P.; Ko, M. G.; Friedrich, P.; Jenkins, M.; Bryan, M. J.; Vegunta, S.; Wittich, C. M.; Lyle, M. A.; Melikian, R.; et al. Speaker Introductions at Internal Medicine Grand Rounds: Forms of Address Reveal Gender Bias. *J Womens Health (Larchmt)* **2017**, *26* (5), 413-419. DOI: 10.1089/jwh.2016.6044.

(23) Madera, J. M.; Hebl, M. R.; Martin, R. C. Gender and letters of recommendation for academia: agentic and communal differences. *J Appl Psychol* **2009**, *94* (6), 1591-1599. DOI: 10.1037/a0016539.

(24) Madera, J. M.; Hebl, M. R.; Dial, H.; Martin, R.; Valian, V. Raising Doubt in Letters of Recommendation for Academia: Gender Differences and Their Impact. *Journal of Business and Psychology* **2019**, *34* (3), 287-303. DOI: 10.1007/s10869-018-9541-1.

(25) Ebbing, D. D.; Gammon, S. D. *General chemistry*, 11^th^ ed.; Cengage, 2017.

(26) *Harvey Itano 1920-2010*. National Academy of Sciences, 2014. http://www.nasonline.org/publications/biographical-memoirs/memoir-pdfs/itano-harvey.pdf (accessed May 26, 2023).
